# Early Intervention With an Ultrapulse CO_2_ Fractional Laser for the Treatment of Traumatic Facial Scars in Children: A Retrospective Study

**DOI:** 10.1111/jocd.70365

**Published:** 2025-07-29

**Authors:** Jingjing Xu, Beibei Niu, Jie Zheng, Wenwen Jiang, Lingdong Zhu

**Affiliations:** ^1^ Department of Plastic and Burn Surgery Children's Hospital Affiliated to Shandong University Jinan Shandong China; ^2^ Department of Plastic and Burn Surgery Jinan Children's Hospital Jinan Shandong China

**Keywords:** children, early treatment, scarring, ultrapulse CO_2_ fractional laser

## Abstract

**Background:**

Ultrapulse CO_2_ fractional lasers are increasingly used for scar treatment. However, the optimal timing for the treatment of traumatic facial scars in children is unclear.

**Aim:**

This retrospective study evaluated the clinical efficacy of ultrapulse CO_2_ fractional laser treatment for immature facial scars in pediatric patients, with a focus on identifying the optimal timing to achieve the best possible outcomes.

**Patients/Methods:**

A total of 106 children with traumatic facial scars were divided into three groups according to when laser treatment was started post‐procedure: 1 month (Group A), 3 months (Group B), and 6 months (Group C). Three months after two treatments, the therapeutic effects, adverse reactions, and satisfaction of the patients' families were compared between the three groups.

**Results:**

After two treatments, the Vancouver Scar Scale (VSS) scores were significantly lower than before treatment in all groups (*p* < 0.001). Pairwise comparisons demonstrated statistically significant differences between Groups A and B, and between Groups A and C (*p* < 0.001). Adverse reactions were not significantly different among the three groups (*p* > 0.05). At the follow‐up after two treatments, significantly more patients were very satisfied in Group A than in Groups B and C (*p* < 0.05).

**Conclusions:**

Early intervention with an ultrapulse CO_2_ fractional laser can effectively treat traumatic facial scars in children. The clinical effect and patient satisfaction were better with treatment initiated 1 month post procedure than with treatment 3–6 months post procedure. It has few adverse reactions and high safety and is worthy of clinical promotion and application.

## 1 Introduction

Scars resulting from burns, trauma, or surgery remain a common clinical challenge and have long been a focus in both plastic dermatology and cosmetic dermatology. Scar formation can negatively affect not only a patient's appearance but also their psychological well‐being and physiological function. In children, who are undergoing active growth and development, the high rate of cellular proliferation and immature skin structure make them more prone to excessive scar formation compared to adults [1]. Moreover, facial scars in children are especially visible and may have long‐term psychosocial impacts.

Conventional scar treatments—such as silicone gel sheeting, pressure therapy, intralesional corticosteroid injection, and surgical revision—have shown certain efficacy, but their clinical outcomes are often limited and inconsistent. In recent years, the emergence of light‐ and energy‐based technologies has provided new opportunities for scar management. Among them, CO_2_ fractional laser technology has been widely adopted for both early‐stage and late‐stage scar modulation, with promising results reported in adult populations. However, the applicability and safety of such treatments in pediatric patients remain less well understood. Children differ significantly from adults in terms of skin structure, pain tolerance, emotional responses, and compliance, which may limit the use of CO_2_ fractional laser in early pediatric scar intervention.

According to expert consensus, most scars gradually transition into the maturation phase between 6 and 12 months after reepithelialization. Active intervention during the first 6 months—when the scar is still immature—is recommended to shorten the unstable remodeling period, promote organized collagen deposition, and prevent the progression to hypertrophic scarring [2]. Despite this, there is a lack of high‐quality clinical data regarding the optimal timing and effectiveness of early laser intervention in children with traumatic scars. To address this gap, the present study aimed to evaluate the clinical efficacy of early intervention using ultrapulse CO_2_ fractional laser for the treatment of immature facial scars in pediatric patients and to explore the optimal window for laser‐based modulation during scar development.

**FIGURE 1:**
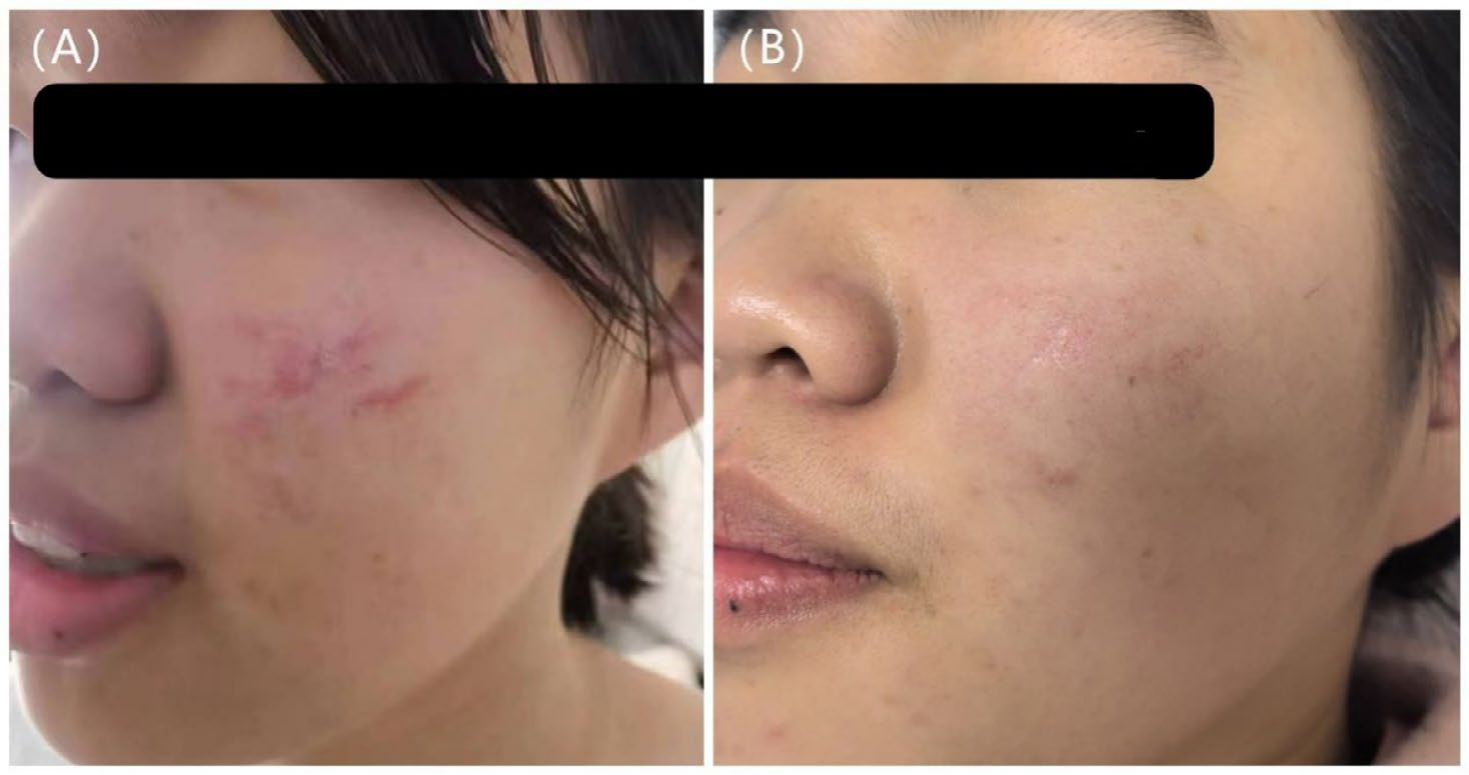
A 14‐year‐old female with a scar on the left side of the face. (A) Scar before initial laser treatment, 1 month post procedure (VSS = 5). (B) 3 months after two treatments with an ultrapulse CO_2_ fractional laser (VSS = 1).

**FIGURE 2:**
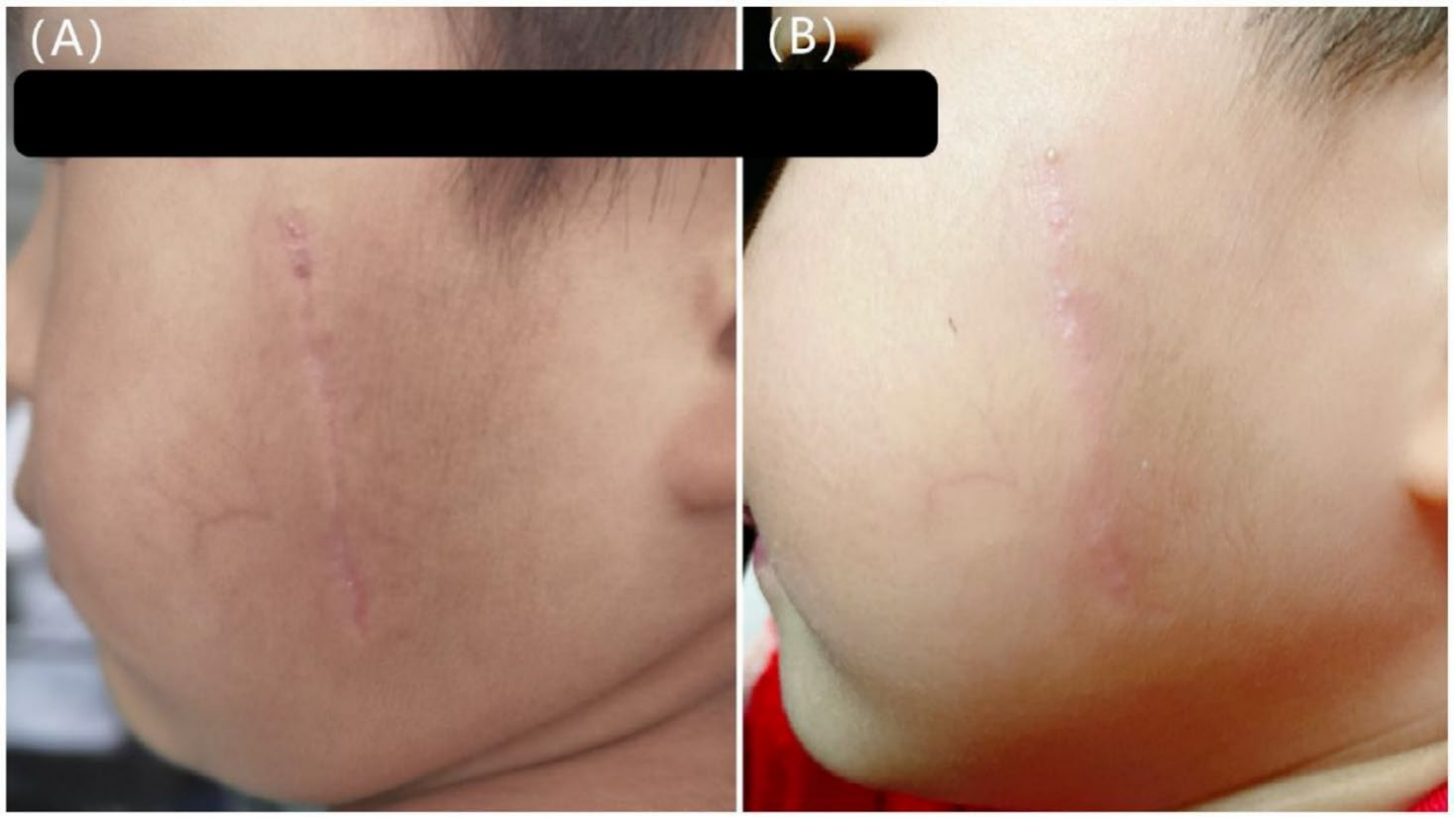
A 3‐year‐old female with a scar on the left side of the face. (A) Scar before initial laser treatment, 3 months post procedure (VSS = 5). (B) 3 months after two treatments with an ultrapulse CO_2_ fractional laser (VSS = 3).

**FIGURE 3:**
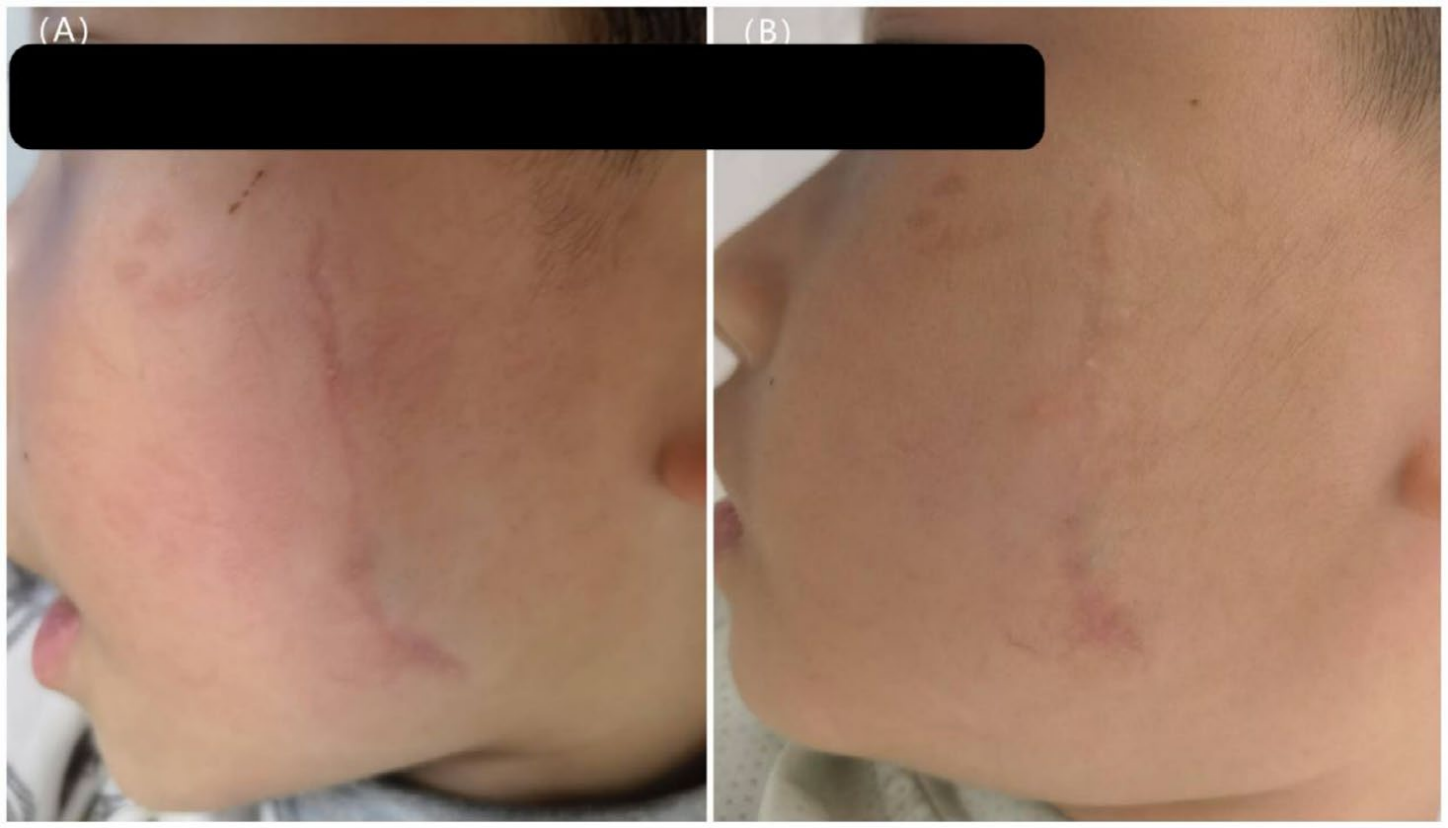
A 4‐year‐old male with a scar on the left side of the face. (A) Scar before initial laser treatment, 6 months post procedure (VSS = 6). (B) 3 months after two treatments with an ultrapulse CO_2_ fractional laser (VSS = 4).

## 2 Materials and Methods

### 2.1 Patient Population

Patients who underwent fine facial sutures at our institution between January 2023 and February 2024 due to facial trauma or skin masses were selected. Scar classification in this study was based on two primary dimensions: maturity (immature vs. mature) and morphology (e.g., linear, disk‐shaped, weblike) [3, 4, 5]. Only immature linear scars were included. Linear scars were selected to ensure consistency in wound type and healing dynamics, thereby minimizing variability related to scar morphology or anatomical distribution. The focus on immature scars was intended to evaluate the early therapeutic effects of CO_2_ fractional laser treatment during the active remodeling phase of scar development.

The inclusion criteria were as follows: (1) ultrapulse CO_2_ fractional laser treatment started 1–6 months after surgery, (2) at least two laser treatments at 2–3‐month intervals, (3) regardless of whether the etiology was traumatic or surgical, all pediatric patients in this study had wounds that extended intraoperatively to the subcutaneous fascial layer, and in some cases, even involved the muscle layer, (4) overall good health.

The exclusion criteria were as follows: (1) Pediatric patients with traumatic or surgical wounds were excluded if the intraoperative skin defect exceeded 1 cm in diameter or if wound closure involved excessive tension, (2) scar ulceration or infection, (3) local dermatitis or allergies, (4) keloids, (5) coagulation dysfunction or immune system abnormalities.

### 2.2 Treatment

An ultrapulse CO_2_ fractional laser (American Lumenis Medical Laser Company) was utilized for treatment.

Treatment methodology was as follows.

#### 2.2.1 Pretreatment Preparation

Prior to CO_2_ fractional laser treatment, the target skin area was thoroughly cleansed. Standardized clinical photographs were taken before each session by the attending physician. All images were captured using the same digital camera (SONY DSC‐WX500) under identical conditions, including background, room setting, lighting, shooting distance, and aperture settings.

After photography, all patients received topical anesthesia with a compound lidocaine cream containing prilocaine (25 mg/g) and lidocaine (25 mg/g). The cream was applied evenly to the treatment area and occluded with plastic wrap for 1 h before proceeding with CO_2_ fractional laser therapy.

#### 2.2.2 Treatment Parameters

Patients were divided into three groups according to the time when laser treatment was initiated after the procedures: 1 month (Group A), 3 months (Group B), and 6 months (Group C). All children were treated with an ultrapulse CO_2_ fractional laser. The DeepFX mode of the device was used to scan the entire scar tissue one time. The treatment energy for Group A was 15–20 mJ/pulse; the treatment energy for Groups B and C was 17.5–22.5 mJ/pulse. Density was set at 5% and the frequency was 300 Hz. The pattern and spot size were set according to the scar shape and area. The treatment endpoints were mild local redness and mild spot exudation. Cold compression was performed for 30 min after treatment. Treatment was performed every 2–3 months, for a total of two treatments. Punctate exfoliation of the epidermis in the scar treatment area was observed immediately after each treatment, and punctate exfoliation of the scabs was observed 7–10 days after treatment. Follow‐up was conducted 3 months after the final treatment session, during which standardized photographs were taken for documentation.

#### 2.2.3 Wound Care

After each treatment, human epidermal growth factor gel was applied for moisturization; subsequent application 2–3 times daily for 1–2 weeks aimed to promote wound healing until the wound scab fell off naturally. Direct water contact with the wound was avoided within 5 days of treatment, and the wound surface was kept clean. Strict sun protection was mandated for 3 months to prevent wound infection and pigmentation. Care was taken not to scratch or press the wound surface. After the wound surface and eschar had completely healed, silicone gel was used.

### 2.3 Outcome Measures

#### 2.3.1 Vancouver Scar Scale (VSS) Scores to Assess Scars [6]

Scar assessments were conducted before treatment and 3 months after the final session by two senior scar specialists who had received standardized training. Evaluations were performed independently in the same examination room under consistent lighting, patient posture, and viewing angle. Clinical assessment followed the VSS, based on both visual inspection and palpation (by applying pressure with the index finger to the center of the scar). Both evaluators were blinded to the treatment groups and did not participate in the intervention process. In cases where the two scores differed, the average value was recorded as the final result.

The VSS was employed to evaluate the scars based on four criteria: pigmentation, thickness, blood supply, and texture. The specific standards for these criteria are as follows:

Pigmentation: scored from 0 to 3, where 0 indicates scar coloration similar to the normal skin of the body; 1 denotes light hyperpigmentation; 2 represents uneven hyperpigmentation; and 3 indicates deep hyperpigmentation.

Thickness: scored from 0 to 4, where 0 represents flatness; 1 indicates a thickness of < 1 mm; a score of 2 denotes 1–2 mm; a score of 3 represents 2–4 mm; and a score of 4 indicates a thickness of > 4 mm.

Blood supply: scored from 0 to 3, where 0 signifies scar coloration similar to the normal skin of the body; 1 indicates a pinkish hue of the scar; 2 denotes a reddish scar color; and 3 represents a purplish scar color.

Texture: scored from 0 to 5, where 0 indicates normal texture; 1 represents softness; 2 denotes deformability under pressure; 3 indicates hardness without deformability under pressure; 4 represents a band‐like texture not affecting movement; and 5 indicates scar contracture affecting movement.

The scores of each item on the VSS are added together, and the maximum total score can reach 15 points. The higher the score, the more severe the scar; the lower the total score, the closer the scar is to normal skin.

#### 2.3.2 Adverse Reactions

Adverse reactions during the entire treatment process, including the number of complications such as skin ulceration, edema, and erythema, pigmentation, hypopigmentation, and aggravation of scars, were recorded and analyzed.

#### 2.3.3 Patient Satisfaction Evaluation

Three months after the two treatments, one family member evaluated the treatment efficacy and satisfaction with their child's scars [7]. The patient satisfaction evaluation scale was used to evaluate the efficacy of the treatment, as follows: dissatisfied (0%–25%), fair (26%–50%), relatively satisfied (51%–75%), and very satisfied (76%–100%).

### 2.4 Statistical Analysis

SPSS22.0 statistical software was used for analysis. Age was expressed as mean ± standard deviation (*x* ± *s*), and the VSS value was expressed as median (M, interquartile range). Rank sum tests (Kruskal–Wallis test and Wilcoxon sign rank test) were used for VSS scores before and after intragroup and intergroup scar treatment, and the chi‐square test was used for the comparison of patient satisfaction and adverse reactions. Statistical significance was set at *p* < 0.05.

## 3 Results

### 3.1 Clinical Characteristics of Patients

This retrospective study included 106 patients (57 males, 49 females) aged 2–12 (5.63 ± 1.53) years. Regarding pathogenic factors, a total of 67 patients presented with facial soft tissue lacerations, while 39 patients underwent surgical excision of benign facial skin lesions, including pilomatricomas, pigmented nevi, and sebaceous nevi. The children were divided into three groups based on the timing of the laser intervention: Group A (*n* = 36) started 1 month post procedure, Group B (*n* = 39) started 3 months post procedure, and Group C (*n* = 31) started 6 months post procedure. Group A comprised 16 males and 20 females, with an average age of 5.72 ± 1.67 years, and pathogenic factors included 23 traumas and 13 surgeries. Group B comprised 20 males and 19 females, with an average age of 5.62 ± 1.46 years, and pathogenic factors included 25 traumas and 14 surgeries. Group C comprised 17 males and 14 females, with an average age of 5.55 ± 1.55 years, and pathogenic factors included 19 traumas and 12 surgeries. The general data such as age, sex, and pathogenic factors were not significantly different among the three groups, reflecting fair comparability (Table 1).

**TABLE 1:** Comparison of general data of three groups of patients.

Group	Number of patients	Sex		Age (*x* ± *s*)	Pathogenic factors (cases)	
		Male	Female		Trauma	Surgery
A	36	20	16	5.72 ± 1.67	23	13
B	39	20	19	5.62 ± 1.46	25	14
C	31	17	14	5.55 ± 1.55	19	12
*χ* ^2^		0.158		N/A	0.070	
*F*		N/A		0.107	N/A	
*p*		0.924		0.898	0.966	

Abbreviation: N/A, not applicable.

### 3.2 VSS Assessment

In the VSS score comparison among the three groups, all VSS scores are reported as the median (Q1, Q3).

#### 3.2.1 Between‐Group Comparison Before Treatment

At baseline, there was a statistically significant difference in VSS scores among the three groups (*H* = 17.204, *p* < 0.001). Specifically, Group B [6 (4, 7)] and Group C [6 (4, 7)] had significantly higher VSS scores than Group A [5 (4, 5)] (*p* < 0.05 for both). This indicates that scars in Groups B and C were more severe prior to treatment compared to Group A.

#### 3.2.2 Within‐Group Comparison Before and After Treatment

Group A (*n* = 36): VSS scores significantly decreased following treatment (pretreatment: 5 (4, 5) vs. posttreatment: 2 (1, 3); *z* = −4.804, *p* < 0.001).

Group B (*n* = 39): A significant reduction in VSS scores was also observed (pretreatment: 6 (4, 7) vs. posttreatment: 4 (3, 5); *z* = −4.582, *p* < 0.001).

Group C (*n* = 31): VSS scores significantly improved after treatment (pretreatment: 6 (4, 7) vs. posttreatment: 4 (3, 5); *z* = −4.707, *p* < 0.001).

These findings demonstrate that CO_2_ fractional laser treatment was effective in reducing scar severity in all three groups (*p* < 0.001).

#### 3.2.3 Between‐Group Comparison After Treatment

After treatment, there were significant differences in VSS scores among the three groups (*H* = 40.312, *p* < 0.001). Group A [2 (1, 3)] had significantly lower posttreatment VSS scores compared to both Group B [4 (3, 5)] and Group C [4 (3, 5)] (*p* < 0.05), indicating a more favorable therapeutic outcome in Group A. There was no statistically significant difference between Groups B and C (*p* > 0.05) (Figures 1, 2, 3).

### 3.3 Adverse Reactions

During treatment, the main adverse reactions were edema and erythema, skin ulceration, and mild pigmentation, which occurred in 0, 1, and 1 case in Group A, respectively; 1, 0, and 0 cases in Group B, respectively; and 0, 0, and 1 case in Group C, respectively. The incidences of adverse reactions in Groups A, B, and C were 5.56% (2/36), 2.56% (1/39), and 3.23% (1/31), respectively (*χ* ^**2**^ = 0.479, *p* = 0.787). Treatment measures for adverse reactions included extending the cold compression time to 2 h for edema, erythema, and external application of human epidermal growth factor gel, which resulted in recovery 2 days later. Skin ulceration was treated with external application of human epidermal growth factor gel and healed in approximately 10 days. Two patients developed mild pigmentation 1.5 months after treatment and were advised to pay more attention to sun protection; their condition improved after 6 months of recovery.

### 3.4 Patient Satisfaction Evaluation

Three months after the two treatments, 52.78% (19/36) of the patients' parents in Group A were very satisfied, which was better than the ratings in Groups B (23.08% [9/39]) and C (19.35% [6/31]) (*p* < 0.05). The rates of very satisfied parents were not significantly different between Groups B and C (*p* > 0.05) (Table 2).

**TABLE 2:** Comparison of patient satisfaction among three groups of patients with scarring after two laser treatments^a^.

Group	Number of patients	Dissatisfied	Neutral	Satisfied	Very satisfied
A	36	1 (2.78)	4 (11.11)	12 (33.33)	19 (52.78)
B	39	4 (10.26)	7 (17.95)	19 (48.72)	9 (23.08)*
C	31	4 (12.9)	6 (19.35)	15 (48.39)	6 (19.35)*
*χ* ^2^ value					10.834
*p* value					0.004

^a^ Data are presented as number of cases (%).

* *p* < 0.05 vs. Group A.

## 4 Discussion

Traumatic scars may result from various causes, including burns, accidental injuries, and surgical procedures. Scar formation is one of the most common sequelae of wound healing and represents a frequent concern in plastic and reconstructive surgery. Clinically, traumatic scars are characterized by abnormal pigmentation, vascular congestion, structural irregularities, and deviation from the contour of surrounding skin—either hypertrophic or atrophic in nature. In addition, some patients experience associated symptoms such as pain and pruritus. Facial trauma is particularly prevalent in pediatric populations. Scarring in this visible area not only compromises aesthetic appearance but can also have a detrimental impact on a child's psychological and emotional development [8]. Therefore, early prevention and timely intervention are of paramount importance in pediatric scar management. The primary therapeutic goals include interrupting the progression of pathological scarring and alleviating subjective symptoms, thereby minimizing both physical and psychosocial burdens on affected children.

Photoelectric treatment of scars has been clinically utilized for many years [9]. Currently, ultrapulse CO_2_ fractional laser is the most commonly used ablative dot matrix laser for scar treatment. It acts on the skin to immediately vaporize the tissue and form small lesions in the surrounding dermis. The collagen of the tissue layer contracts upon heating, stimulating the tissue to controllably repair the new damage and improve the scars [10, 11, 12, 13]. Children's skin is still in the developmental stage, characterized by a thinner epidermis, immature barrier function, and a high rate of cellular metabolism and tissue regeneration. These physiological features make pediatric skin more responsive—and potentially more sensitive—to laser therapy compared to adult skin. This heightened sensitivity carries a dual implication. On the one hand, the strong regenerative capacity of pediatric tissue may enhance the effectiveness of fractional laser treatment by promoting the remodeling of scar tissue into structures more similar to normal skin, including normalization of collagen composition [14, 15, 16, 17]. On the other hand, the incomplete development of dermal structures and higher water content in pediatric skin may increase the risk of laser‐associated complications if parameters are not carefully adjusted. These risks include post‐inflammatory hyperpigmentation, hypopigmentation, delayed wound healing, or even pathological scar formation such as hypertrophic scars [2, 18]. Therefore, laser treatment for pediatric scars must be approached with particular caution, emphasizing individualized parameter settings and meticulous technique. Especially during early scar formation, low initial energy levels are recommended to minimize unnecessary thermal damage [19]. Children, especially young children, have a low pain tolerance and can display poor cooperation regarding facial wound treatment. Therefore, administering local skin anesthesia to children is necessary to improve the treatment experience.

Previous studies have demonstrated that within the first month after surgery, scar thickness following plastic and tension‐reducing sutures typically remains below 3 mm, with a relatively low density of newly formed capillaries in the granulation tissue. At this early stage, the application of ultrapulse CO₂ fractional laser—delivered in DeepFX mode—can reach the dermal layer and induce beneficial molecular changes. These include increased expression of heat shock protein 70 (HSP70) and matrix metalloproteinases (MMPs), downregulation of transforming growth factor‐beta 1 (TGF‐β1), reduction in the collagen I/III ratio, and enhanced collagen remodeling. Collectively, these effects promote the regeneration of skin tissue and improve scar quality [20, 21, 22]. In the present study, patients in Group A received ultrapulse CO₂ fractional laser treatment within 1 month of injury using DeepFX mode at an energy setting of 15–20 mJ/pulse. This early intervention effectively disrupted the pathological cascade of scar hyperplasia and facilitated the remodeling of scar tissue into skin‐like structures. The posttreatment VSS scores in Group A were significantly reduced compared to both their baseline values and those of Groups B and C, who underwent treatment 3–6 months after injury. Moreover, most parents of Group A patients reported higher satisfaction with clinical outcomes compared to parents in the later treatment groups. The incidence of adverse reactions did not significantly differ among the three groups. Scars typically enter a rapid proliferative phase between 2 and 3 months post injury, during which time they thicken and harden due to dense collagen deposition. Initiating laser treatment at this stage may lead to reduced efficacy, as collagen remodeling becomes more difficult and multiple treatment sessions may be required. In contrast, early low‐energy intervention promotes skin regeneration, mitigates adverse scar development, and better preserves both function and appearance in pediatric patients. Early treatment may also help to minimize the psychological burden of visible facial scarring on children and their families.

In this study, although the etiologies of traumatic and surgical wounds differ, our strict inclusion criteria—specifically selecting cases with small defect size and low‐tension primary closure—ensured a high degree of homogeneity between the two groups. As a result, scars in both groups were comparable in depth, morphology, and healing conditions. No significant differences in clinical response to CO_2_ fractional laser treatment were observed. Despite the comparable outcomes, potential mechanistic differences may still exist due to variations in wound biology, such as local inflammatory responses, collagen remodeling patterns, and tissue vascularization between traumatic and surgical scars. These subtle biological distinctions may not have been fully captured in our current study but could influence treatment outcomes in more heterogeneous patient populations. Therefore, the importance of individualized laser parameter adjustment and treatment planning should be emphasized—tailored not only to scar maturity and location but also to the underlying etiology and tissue characteristics.

In this clinical investigation, early intervention using an ultrapulse CO_2_ fractional laser achieved promising preliminary outcomes in the treatment of immature facial scars in children. Most patients experienced marked improvement in scar appearance and texture. However, several practical challenges were noted. Due to limited cooperation, some younger children had difficulty maintaining a stable posture during the procedure, potentially compromising treatment accuracy. Additionally, two cases of mild post‐inflammatory hyperpigmentation were observed, both associated with inadequate sun protection posttreatment. One child developed skin ulceration following treatment, primarily due to accidental removal of the scab at home, which resulted in wound oozing and delayed healing. These events underscore the importance of not only selecting appropriate laser parameters but also ensuring meticulous posttreatment skin care and caregiver education to prevent complications and optimize outcomes in pediatric patients.

This study has several limitations. First, the retrospective design limits control over confounding variables, and potential selection or observer bias cannot be entirely excluded, despite the use of strict inclusion criteria and standardized assessments. The primary outcome, evaluated using the semi‐subjective VSS, may also be influenced by evaluator bias. Second, the single‐center setting and focus on immature linear facial scars may limit the generalizability of the findings to other scar types or anatomical regions. Third, the relatively small sample size and short follow‐up period may not fully capture long‐term treatment effects, especially given the variability of scar maturation in children. Future prospective, multicenter studies with longer follow‐up and more objective evaluation tools—such as ultrasound imaging and patient‐reported outcomes—are warranted to validate and expand upon these results.

In summary, early intervention with an ultrapulse CO_2_ fractional laser for the treatment of traumatic facial scars in children has significant clinical effects. Patients who received laser treatment at 1 month post procedure showed superior scar improvement and higher parental satisfaction compared to those treated at 3–6 months. The procedure was associated with a low incidence of adverse effects, supporting its high safety profile. These findings support the clinical value of early laser intervention in pediatric scar management and suggest its potential for broader application and clinical promotion.

## Author Contributions

J.X. conceived and designed the study, as well as wrote the manuscript. J.X., J.Z., and B.N. provided study materials and patient data. W.J. collected and assembled the data. J.X. and B.N. conducted data analysis and wrote the results. L.Z. was responsible for reviewing and revising the manuscript.

## Ethics Statement

This study was approved by the Ethics Committee of Children's Hospital affiliated to Shandong University (No: SDFE‐IRB/T‐2025046).

## Consent

Informed consent and permission for the publication of medical images were obtained from the patients.

## Conflicts of Interest

The authors declare no conflicts of interest.

## Data Availability

The data that support the findings of this study are available from the corresponding author upon reasonable request.
